# Moisture-responsive films of cellulose stearoyl esters showing reversible shape transitions

**DOI:** 10.1038/srep11011

**Published:** 2015-06-08

**Authors:** Kai Zhang, Andreas Geissler, Michaela Standhardt, Sabrina Mehlhase, Markus Gallei, Longquan Chen, Christina Marie Thiele

**Affiliations:** 1 Ernst-Berl-Institute for Chemical Engineering and Macromolecular Science, Technische Universität Darmstadt, Alarich-Weiss-Str. 8, D-64287 Darmstadt, Germany; 2 Clemens-Schöpf-Institut für Organische Chemie und Biochemie, Technische Universität Darmstadt, Alarich-Weiss-Str. 16, D-64287 Darmstadt, Germany; 3 School of Mechanics and Engineering, Southwest Jiaotong University, 610031, Chengdu Chine.

## Abstract

Moisture-responsive materials are gaining greater interest for their potentially wide applications and the readily access to moisture. In this study, we show the fabrication of moisture-responsive, self-standing films using sustainable cellulose as starting material. Cellulose was modified by stearoyl moieties at first, leading to cellulose stearoyl esters (CSEs) with diverse degrees of substitution (DSs). The films of CSE with a low DS of 0.3 (CSE_0.3_) exhibited moisture-responsive properties, while CSEs with higher DSs of 1.3 or 3 (CSE_1.3_ and CSE_3_) not. The CSE_0.3_ films could reversibly fold and unfold as rhythmical bending motions within a local moisture gradient due to the ab- and desorption of water molecules at the film surface. By spray-coating CSE_3_ nanoparticles (NPs) onto CSE_0.3_ films, moisture-responsive films with non-wetting surface were obtained, which can perform quick reversible bending movements and continuous shape transition on water. Furthermore, bilayer films containing one layer of CSE_0.3_ at one side and one layer of CSE_3_ at the other side exhibited combined responsiveness to moisture and temperature. By varying the thickness of CSE_0.3_ films, the minimal bending extent can be adjusted due to altered mechanical resistances, which allows a bending movement preferentially beginning with the thinner side.

Moisture-responsive behaviors are widely spread in nature, such as moisture-responsive events of plants and fungi during the dispersion of seeds and spores^1^,^2^, the opening of pine cones, twisting and bending of wheat awns (*Triticum turgidum*)^3^,^4^,^5^,^6^. During the alteration of the environmental moisture content, i.e. relative humidity, a particular part of the biological systems reversibly absorbs or releases the moisture. During this process, a mechanical deformation takes place, with the goal to perform a desired function such as directed complex motions^1^,^4^. Inspired by nature, moisture-responsive materials have awoken great interest. Due to the environmentally friendly character of water vapor and its easy accessibility, moisture-responsive materials are promising candidates for broad applications, e.g. sensors^7^,^8^, actuators^9^,^10^,^11^ or construction of soft robots^12^,^13^.

Although water- or moisture-responsive polymers are readily to be prepared, the polymers that can be transformed into functional, fast responsive materials beyond the molecular level are still limited. Films from polyurethane or cross-linked chitosan with an epoxy compound have shown moisture-responsive properties^14^,^15^,^16^. Water-responsive hydrogels based on polyglycidyl methacrylate have been used for the construction of responsive actuator^17^. Most recently, the composite films consisting of polypyrrole and polyol-borate were shown to be fast moisture-responsive and of particular interest for generation of piezoelectric energy^11^.

In addition, there is ever greater interest of using sustainable compounds, e.g. using cellulose, for the construction of functional materials in recent years^18^,^19^,^20^. Cellulose, consisting of β–1,4–linked anhydroglucose units (AGUs), represents the most abundant material on earth. Cellulose esters and ethers have found many applications in our daily life, such as textiles, food additives and packaging materials^20^,^21^. Moisture-responsive, shape-memory composites containing cellulose nanowhiskers or microcrystalline cellulose have been reported recently^22^,^23^,^24^,^25^,^26^,^27^. In addition to crystalline cellulose as reinforcing component, a synthetic polymer, such as ethylene oxide-epichlorohydrin copolymer (1:1), poly(D, L-lactide), polyurethane or poly(glycerol sebacate urethane), was generally required as the responsive components in these composites^15^,^22^,^23^,^24^,^25^,^26^,^27^. In contrast, still no successful fabrication of moisture-responsive devices from pristine cellulose-derived materials without any other additives has been reported, such as self-standing films. This fact on the one hand limits the application of cellulose and on the other hand addresses new challenges for the development of cellulose-based compounds, which requires the precise control on polar and non-polar moieties.

In this report, we show the first moisture-responsive, self-standing and transparent films using derivatives of sustainable cellulose, cellulose stearoyl esters (CSEs). Thin films of CSE with a low degree of substitution (DS) of 0.3 (CSE_0.3_) showed a fast and reversible response to moisture. In contrast, thin films from CSE with higher DS of 1.3 (CSE_1.3_) and 3 (CSE_3_) did not show significant moisture-response. Moreover, CSE_0.3_ films were converted into non-wetting films after spray-coating with nanoparticles (NPs) from CSE_3_, which allowed CSE_0.3_ films to continuously move on water surface. By combining the film of CSE_0.3_ and CSE_3_, bilayer films containing a hydrophilic and a hydrophobic layer at each side were further prepared, which are responsive to both temperature and moisture.

## Results

### Synthesis of cellulose stearoyl esters

**Figure 1:**
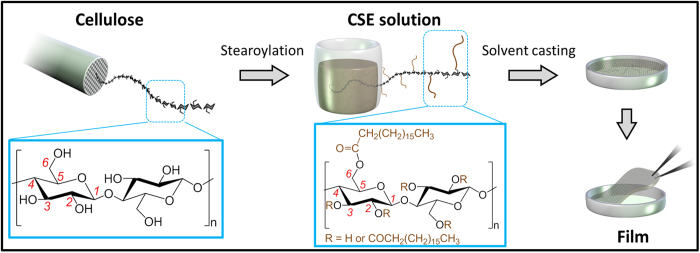
Schematic representation for the synthesis of cellulose stearoyl esters (CSEs) from cellulose and the fabrication of films by solvent-casting solutions of CSEs.

**Figure 2:**
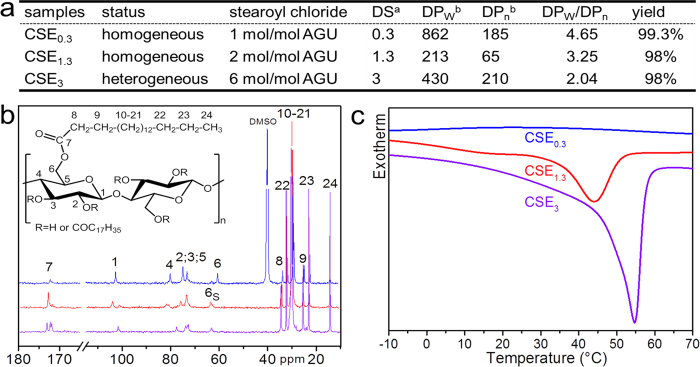
Cellulose stearoyl esters (CSEs). (**a**) Synthesis and characterization of CSEs. ^a^DSs were determined via elemental analysis. ^b^Weight- and number-averaged degrees of polymerization (DP_W_ and DP_n_). The DP of CSE_0.3_ was measured due to its solubility in DMF/LiCl solution by using a RI detector. (**b**) ^3^C NMR spectra (180–10 ppm) of CSEs with different DSs recorded in corresponding solvents at 50 °C: CSE_0.3_ in dimethyl sulfoxide (DMSO)-d_6_ (blue); CSE_1.3_ in pyridine-d_5_ (red) and CSE_3_ in benzene-d_6_ (purple). The inset shows the schematic chemical structure of CSE. (**c**) Differential scanning calorimetry (DSC) curves (2nd cycle) of CSE_0.3_, CSE_1.3_ and CSE_3_.

The primary concept for the fabrication of stimuli-responsive films using cellulose stearoyl esters (CSEs) is shown in Fig. 1. Cellulose stearoyl esters (CSEs) with different degrees of substitution (DSs) of 0.3, 1.3 and 3 were synthesized via two distinct synthesis routes, either heterogeneously with cellulose suspended in pyridine or homogeneously with cellulose dissolved in DMAc/LiCl before the chemical modification (Scheme S1). The DS of CSE could be adjusted by varying the amount of acid chloride for the esterification (Fig. 2a)^28^,^29^. CSE_3_ with the maximal DS of 3 was achieved with 6 mol stearoyl chloride/mol AGUs, while cellulose underwent a progress from a heterogeneous to a homogeneous condition during the reaction. CSEs with lower DS of 1.3 and 0.3 were synthesized with cellulose dissolved in DMAc/LiCl before the reaction using 2 and 1 mol stearoyl chloride/mol AGU, respectively. Although the synthesis of CSE_1.3_ in DMAc/LiCl begins with dissolved cellulose, it ends up heterogeneously due to the poor solubility of obtained CSE_1.3_ in DMAc/LiCl. FTIR spectra of synthesized CSEs showed typical signals attributed to aliphatic chains and ester bonds (Figure S1). All signals attributed to stearoyl groups exhibit increasing intensities with higher DS, while the intensity of the FTIR band attributed to stretching vibrations of hydroxyl groups decreases and the peak maximum is shifting to higher wavenumbers.

In comparison to widely used ^1^H NMR and solid-state ^13^C CP/MAS NMR spectroscopy, liquid-state ^13^C NMR and 2D NMR spectra of long chain fatty acid esters of cellulose with low and intermediate DS are scarcely performed in contrast to cellulose esters with short alkane chains (<6 carbons)^30^,^31^,^32^,^33^,^34^. For the liquid-state NMR analysis of CSEs as well as the solvent-casting process, suitable solvents were chosen based on their solubility parameters (Table S1-S3): benzene-d_6_ for CSE_3_, pyridine-d_5_ for CSE_1.3_ and DMSO–d_6_ for CSE_0.3_.

By using 2D ^1^H,^1^H-correlation spectroscopy (COSY), heteronuclear single-quantum correlation (HSQC) and heteronuclear multi-bond correlation spectroscopy (HMBC), the exact assignment of the signals was performed (Figure S2–S4). Representative ^13^C NMR spectra of CSEs with different DSs are shown in Fig. 2b. The signals around ~173 ppm are attributed to the carbon of C = O groups. The signals between 60 and 10 ppm are ascribed to the carbons of aliphatic chains, while the carbons of AGUs of cellulose represent signals between 110 and 60 ppm (Table S4)^31^,^35^,^36^. The shift of C6-signal from 60.2 to ~63 ppm indicates the esterification of primary hydroxyl groups. The splitting of C1-signal with the emergence of a new signal at 101.5 ppm is caused by the derivatization of hydroxyl groups on C2-position. It is visible that the CSE_0.3_ exhibits only a partial shift of the C6-signal, while the C6-signals of CSE_1.3_ and CSE_3_ are totally shifted from 60 to ~63 ppm. Thus, the primary hydroxyl groups in CSE_0.3_ were only partially modified by stearoyl groups, while those of CSE_1.3_ and CSE_3_ were totally esterified. Moreover, the C1-signal within the NMR spectrum of CSE_0.3_ was not shifted, implying no modification of hydroxyl groups at C2–position. In comparison, CSE_1.3_ and CSE_3_ exhibited partial and total derivatization of hydroxyl groups at C2-positions, according to the splitting of C1-signal and total shift of C1-signal, respectively^30^. Furthermore, in HMBC spectrum of CSE_0.3_, the ^2^J and ^3^J couplings of the ester carbon at C6-position (C = O_@6_) with the hydrogen atoms at C8 and C9 in aliphatic chains are notable (Figure S4a).

The presence of diverse contents of stearoyl moieties is also represented by DSC measurements (Fig. 2c). No significant crystalline character is observable for CSE_0.3_ due to very low content of stearoyl groups. In contrast, CSE_3_ shows a strong DSC signal with the maximum at 55 °C, indicating the presence of crystalline structure that was constructed by stearoyl groups. CSE_1.3_ with a DS of 1.3 shows a glass-transition temperature at ~10 °C and a broad peak with a maximum at 44 °C. The shape of the peaks ascribed to crystalline character is typical for partially crystalline polymers. As reported before, highly substituted cellulose long chain esters with aliphatic chain lengths of more than 12 are able to form ordered regions via side chains^37^,^38^. Hence, the aliphatic chains at cellulose backbones should have partially crystallized. The extent of the crystalline regions increases with higher content of stearoyl groups, based on the shifted peak maximum to higher temperature and stronger peak intensity.

### Fabrication and characterization of films

**Figure 3:**
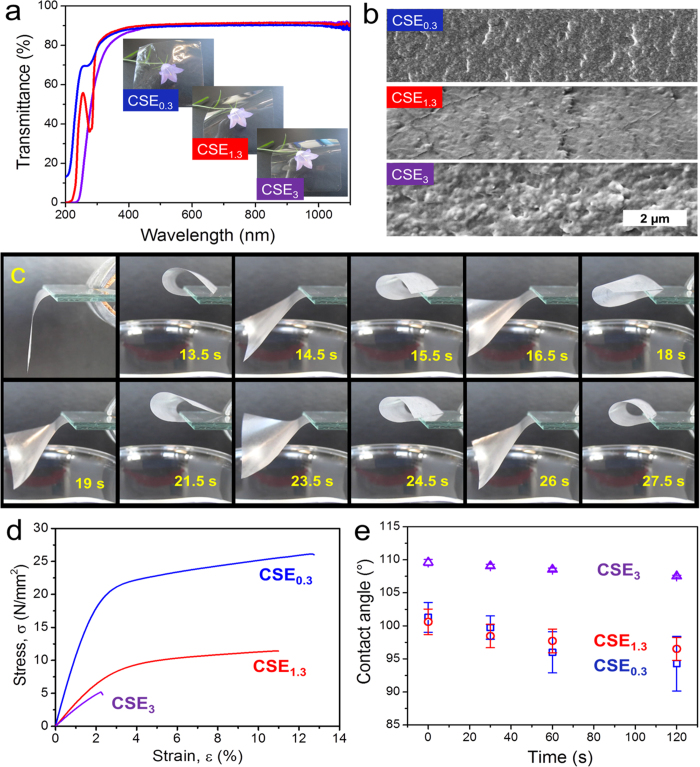
Characteristics of CSE_0.3_, CSE_1.3_ and CSE_3_ films with a thickness of 21.2 ± 1.6, 20.6 ± 1.6 and 20.3 ± 2.2 μm, respectively. (**a**) UV-Vis transmittance spectra and representative photographs of CSEs films (40 × 40 mm^2^) covering a flower for the visualization of their transparency. (**b**) SEM images of the cross sections of CSEs films with scale bar of 2 μm. (**c**) Snapshots of a bending CSE_0.3_ film (40 × 40 mm^2^) with a thickness of 21.2 ± 1.6 μm. One side of the film is fixed between two glass slides. Movie SM1 was recorded at 35 ± 2% RH and 22 ± 3 °C. The moisture-responsive movements immediately took place after placing warm water of 37 °C under the film. (**d**) Representative stress-strain curves recorded during tensile strength tests on the CSEs films at 23 ± 1 °C and 50 ± 2% RH. (**e**) Static water contact angles on CSEs films at 23 ± 1 °C and 50 ± 2% RH.

After solvent-casting solutions of CSEs, films with a thickness of around 20 μm are all highly transparent (Figs 1,3a & S5). The transmittance for visible light within the wavelength range of 400–800 nm is constantly around 90%. Moreover, the films contain homogeneous structure as shown by SEM images of their cross sections (Fig. 3b). In comparison, the membrane of regenerated cellulose is also highly transparent, but shows a layered structure (Figure S6). The homogeneous structure of CSEs films is ascribed to the drying process from their solutions.

Among the CSEs films, only CSE_0.3_ films showed the most pronounced moisture-responsive motions. CSE_0.3_ films bend when they are exposed to water vapor under ambient conditions of 35 ± 2% relative humidity (RH) and 22 ± 3 °C (Fig. 3c, Movie SM1). In comparison, the films from CSE_1.3_ and CSE_3_ did not show significant response when exposed to water vapor under the same conditions (Figure S7). After contacting with water vapor, the CSE_0.3_ strip began to bend and fold up within 1–2 s (Fig. 3c). After reaching the maximal bending extent, the strip bent down within 1–2 s (Movie SM1). Once it has contact with water vapor, the CSE_0.3_ strip could curl up again and this folding-unfolding process repeat rhythmically with the same frequency. In contrast, a cellulose membrane with a comparable thickness (24.3 ± 1.2 μm) needed much longer time (~10 s) to fully bend (Movie SM2). No responsive bending of CSE_0.3_ films were observed by approaching them to silicone oil of 37 °C (Figure S8). Thus, a heating effect, i.e. the temperature (37 °C), can be excluded as trigger for the movements of CSE_0.3_ films above the warm water surface. The fast movements in response to moisture allow such films to be promising candidates for energy harvesting, such as generator for piezoelectricity^11^,^39^,^40^. Moreover, they can be used as substrates for the embedded sensors for moisture or even as prototypes for the development of artificial skin^41^,^42^. Thus, it is essential to understand the properties of films based on esterified celluloses and to find out the mechanism for the rapid responsiveness.

The mechanical properties of CSEs films were further studied by measuring their tensile strengths (Fig. 3d). CSE_0.3_ films of ~20 μm showed the highest tensile strength and elastic modulus among the CSEs films. With an increasing DS from 0.3 through 1.3 to 3, steadily lower tensile strengths and elastic modulus were determined for CSE_1.3_ and CSE_3_ (Fig. 3d & Table S5). Moreover, the CSE_0.3_ film exhibits a fracture strain, i.e. strain at break, of 12.7% ± 1.7%, in comparison to 10.7% ± 2.3% and 2.5% ± 1% of CSE_1.3_ and CSE_3_ films, respectively. Thus, CSE_0.3_ film is the strongest and at the same time the most flexible one among all three kinds of CSEs films. Nevertheless, the tensile strength and elastic modulus of CSE_0.3_ films are much lower than those of cellulose membranes (Table S5 & Figure S9). However, the cellulose membrane is very stiff, so that a deformation is difficult (Movie SM2). Thus, a low amount of stearoyl groups at cellulose backbone dedicate themselves as plasticizer within CSE_0.3_ films^43^. Furthermore, all three CSEs films exhibited hydrophobic surfaces with static water contact angles of >90° (Fig. 3e), which are ascribed to enhanced non-polarity due to the presence of stearoyl groups.

**Figure 4:**
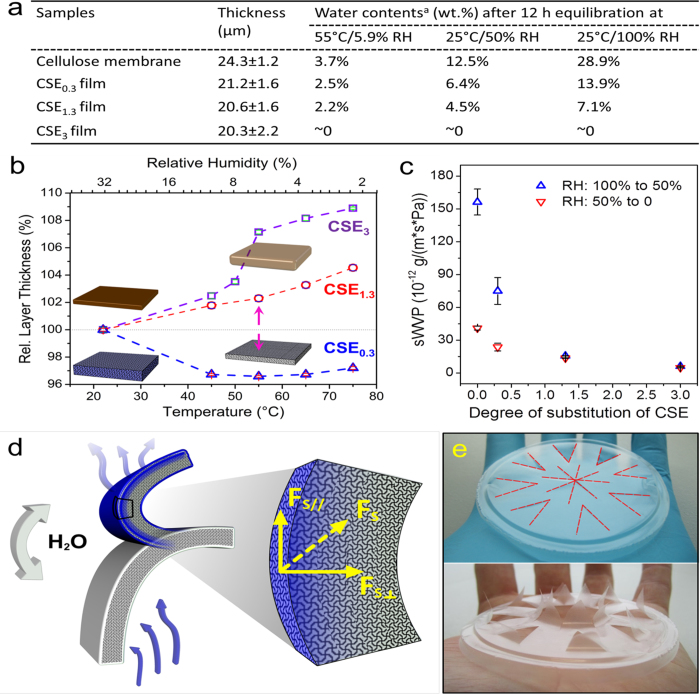
(**a**) The water contents in CSEs films after the equilibration under different temperatures and RH. A membrane from regenerated cellulose with the thickness of 24.3 ± 1.2 μm was analyzed as reference. ^a^ Standard deviations for all water contents are <5%. (**b**) Temperature- and RH-dependent alteration of thicknesses of CSEs films measured by ellipsometry. The short green, black and magenta lines are error bars. The magenta arrows indicate the thickness change from the initial film thickness, which was normalized as 100%. (**c**) Static water vapor permeability (sWVP) of a cellulose membrane (with a DS of 0) and CSEs films. The difference of water vapor partial pressure between the two sides of the membranes and films was 1.4 kPa. The short black lines are error bars. (**d**) Schematic representation for the moisture-responsive bending of CSE_0.3_ film, which is triggered by water absorption and desorption. The blue layer indicates the surface layer of the film with absorbed water. The white layers represent the surface layer of the film without water. (**e**) Photo images of a moisture-responsive CSE_0.3_ film with a thickness of 19.9 ± 1.2 μm and pre-cut triangle openings by placing the film on a hand with and without a rubber glove. The pre-cut positions are marked by red dotted lines.

In addition to the mechanical properties, interactions between CSEs films and water are further analyzed regarding the moisture-responsiveness of CSE_0.3_ films. The capability of binding water at diverse RH was evaluated by measuring the amount of absorbed water by CSEs films (Fig. 4a & S10). Under a certain RH and temperature, CSE_0.3_ film can absorb more water than films of CSE_1.3_, but less than cellulose membrane. For instance, after the equilibration in the environments with a RH of 100% at 25 °C, it is visible that CSE_0.3_ films absorbed up to about 14 wt.% water. In comparison, CSE_1.3_ films only contained 7.1 wt.%, while CSE_3_ did not show significant absorption of water^44^. At a lower humidity of 50% RH at 25 °C, CSE_0.3_ film contains 6.4 wt.% water, and even less water (2.4 wt.%) is found at 5.9% RH. The feasibility of binding water is primarily due to the presence of numerous of hydroxyl groups within CSE_0.3_ and CSE_1.3_ films. Thus, at a low humidity, e.g. ambient humidity of 35%, CSE_0.3_ films are still capable of binding more water, if they are exposed to water vapor with higher contents of moisture.

After the absorption of water molecules, CSE_0.3_ films are swollen, which is represented by the increase of film thicknesses as detected by ellipsometry (Fig. 4b). By decreasing the temperature from 55 °C to 22 °C and thus increasing the RH from 5.9% to 35%, a thickness increase of 3.5% was measured for CSE_0.3_ films. This thickness increase is primarily caused by water absorption during the rising of RH. Moreover, the thickness alteration is reversible, indicating that the water molecules in CSE_0.3_ films are releasable and CSE_0.3_ films can rebind water molecules. In comparison, the thickness of CSE_1.3_ and CSE_3_ films decreased during the same treatment for 2% and 7.1%, respectively (Fig. 4b). Because the films for ellipsometry analysis exhibited a relative large surface area (20 × 20 mm^2^) and a much lower thickness (~180 nm), the decrease of the thickness represents the shrinkage of the film volume. The reduction of the volumes of CSE_1.3_ and CSE_3_ films at 22 °C is ascribed to the presence of a partially crystalline structure and thus a more compact structure. At the temperature of higher than 55 °C (Fig. 2c), a larger volume is resulted due to the formation of disordered structures within CSE_1.3_ and CSE_3_ films. Hence, CSE_1.3_ and CSE_3_ films show temperature-responsive property, which is based on the construction and destruction of crystalline regions consisting of stearoyl moieties. This temperature-responsive behavior can be represented by reversible changes of film volumes.

The feasibility of CSEs films to absorb and desorb water was further represented by their static water vapor permeability (sWVP). As shown in Fig. 4c, CSE_0.3_ films are more permeable for water vapor than CSE_1.3_ and CSE_3_ films. Under equal conditions, the sWVP is strongly affected by the swelling ability of a film. CSE_0.3_ films exhibit a significantly higher sWVP than CSE_1.3_ and CSE_3_ films, because CSE_0.3_ films are more swellable due to the presence of more hydroxyl groups. Moreover, sWVP of CSE_0.3_ films is affected by the moisture in the environment. Under higher humidity, CSE_0.3_ films contain more water and their sWVP is higher, as shown by the water permeation process from 100% RH to 50% RH in comparison to the same process from 50% RH to ~0% RH. Thus, CSE_0.3_ films can not only absorb and desorb water molecules, but also are permeable to water molecules. The content of water within CSE_0.3_ films is adjusted by the humidity of the environment. When a CSE_0.3_ film is exposed to water vapor, it absorbs water molecules at the surface facing the water vapor. The film expands vertically and horizontally, which causes a vertical and a horizontal swelling force. As the result, a net folding force, the swelling force *F*_*S*_, is generated. It applies on the film and causes the film to bend (Fig. 4d).

During the film deformation, the elastic energy is increased at the cost of the mechanical energy caused by the swelling force. For a thin film, the bending energy can be estimated as *Bk*^*2*^*L*^*2*,^^45^, where *k* is the curvature, *L* is the characteristic length of the bending (in the order of the maximum bending radii) and *B* = *Eh*^3^/[12×(1 − *v*^2^)] is the bending stiffness with *E*: the elastic modulus, *h*: the thickness of the film and *v*: the Poisson’s ratio (~0.3 for microcrystalline cellulose)^46^. The mechanical energy scales as *F*_*S*_*L*. By balancing these two terms, *F*_*S*_ *~* *Bk*^*2*^*L* is obtained. Considering the characteristic parameters of CSE_0.3_ films, *E* = 1118 MPa, *h* = 21.2 μm, *L* = 4.6 mm and *k* ~ *1/L*, a folding force of *F*_*S*_ ~ 16 μN is obtained, which is comparable to the force to bend stiff cantilevers^47^.

After folding up, the swollen surface of the CSE_0.3_ film is now in an environment with lower RH, i.e. lower moisture content. Therefore, water molecules quickly evaporate, leading to the release of the bending force. Then, the CSE_0.3_ film unfolds and falls down under the gravity to its initial state (Movie SM1). By absorbing and desorbing water, the CSE_0.3_ film can reversibly fold up and fall down, i.e., the CSE_0.3_ film shows a moisture-responsive and shape-memory property. The moisture-responsiveness of the CSE_0.3_ film is so sensitive that even human skin can induce responsive movements, as shown by the opening of the pre-cut triangles within the film (Fig. 4e).

### Moisture-responsive CSE_0.3_ films with modified properties

**Figure 5:**
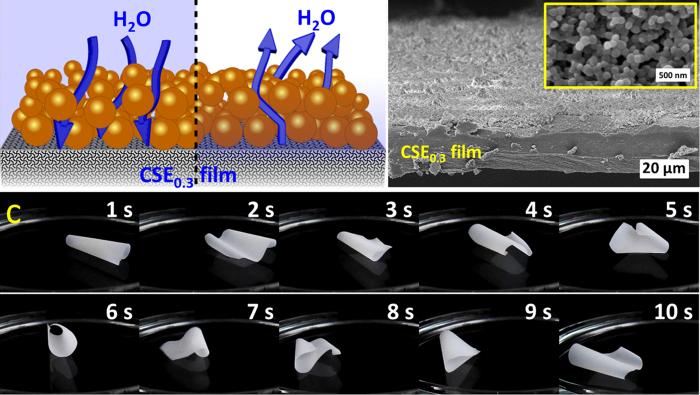
(**a**) Schematic representation for the CSE_0.3_ film covered with CSE_3_ NPs. Under conditions with high moisture contents (left side with blue background), water vapor can penetrate through the NPs layer and reach CSE_0.3_ film (represented by blue arrows). Under conditions with low moisture contents (right side), water vapor can be emitted again (represented by blue arrows). (**b**) A SEM image of the side profile of a CSE_0.3_ film with CSE_3_ NPs on the surface. Scale bar: 20 μm. The inset shows the SEM image of the CSE_3_ NPs with the scale bar of 500 nm. (**c**) Snapshots of CSE_0.3_ films with homogeneous thickness of 21.2 ± 1.6 μm coated with CSE_3_ NPs floating and moving on water surface at 22 °C in the air (Movie SM3).

Moisture-responsive CSE_0.3_ films can be further modified into non-wetting films or multi-responsive films by combining NPs or films of CSE_3_. Although the film from CSE_3_ did not show moisture-responsive motions because of its high amount of non-polar stearoyl groups, CSE_3_ can be transformed into NPs via nanoprecipitation. CSE_3_ NPs can be used for the fabrication of superhydrophobic surfaces, if they are spray-coated onto diverse substrates^44^. Due to the fast moisture-responsive movement of CSE_0.3_ film, it is of great interest to fabricate non-wetting CSE_0.3_ films for the applications even in the presence of high amounts of liquid water. To achieve this goal, CSE_0.3_ films were covered with CSE_3_ NPs through spray-coating, leading to a non-wetting NP layer attached at the film surface (Fig. 5a,b). The NPs from CSE_3_ exhibit an average diameter of 98.8 ± 30 nm based on the diameters of 100 single NPs. The sprayed layer has an average thickness of 2.3 ± 1.4 μm based on SEM measurements and the density of NPs was measured to be ~0.34 mg/cm^2^ film. As-prepared CSE_0.3_ films covered with NPs showed continuous bending movements on water surface at 22 °C (Fig. 5c & Movie SM3). Thus, the layer of CSE_3_ NPs is non-wetting and is permeable for water vapor, which reaches the surface of CSE_0.3_ films and induces the reversible moisture-responsive movement of CSE_0.3_ films (Fig. 5c). Moreover, during the bending movements on water, the shape deformation and transition of CSE_0.3_ films were totally reversible.

**Figure 6:**
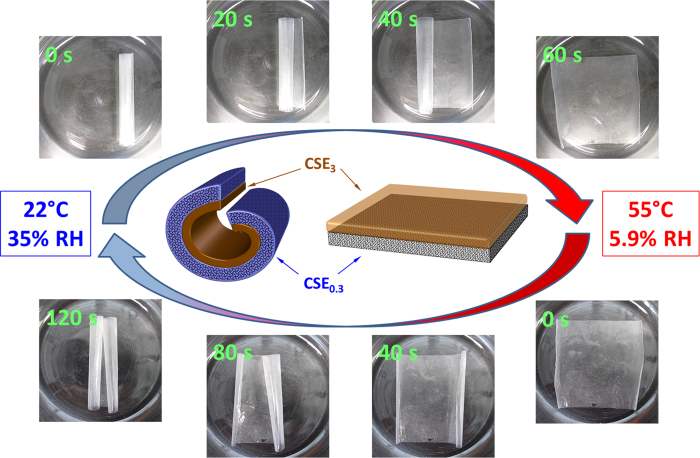
Snapshots captured after distinct times showing the curling and uncurling of a bilayer film (40 × 40 mm^2^) by altering the environmental conditions. A schematic sketch of the two main states of the bilayer film is shown in the center. On the left side: the curled bilayer film consisting of solidified CSE_3_ layer at 22 °C and curled CSE_0.3_ layer due to high moisture content (35% RH). On the right side: flat bilayer film consisting of molten CSE_3_ layer at 55 °C and flat CSE_0.3_ layer due to low moisture content (5.9% RH).

Moreover, the film of CSE_3_ is sensitive to temperature and undergoes a temperature-induced volume expansion (Fig. 4b). By combining a CSE_3_ film of ~20 μm with a moisture-responsive CSE_0.3_ film of ~20 μm, bilayer films were obtained which show a hydrophilic surface at one side and a hydrophobic surface at the other side (Fig. 6). These bilayer films combine the responsive properties of both components. As shown in Fig. 6, as long as a bilayer film was kept under the conditions for its formation (55 °C and 5.9% RH), they stayed as planar films. By cooling down to 22 °C with the accompanied increase of RH to 35%, the bilayer film started to curl due to the solidification and slight contraction of CSE_3_ film. Finally, the bilayer film rolls up and forms a tight roll with CSE_0.3_ at the outside. By placing the film back to the condition of 55 °C and 5.9% RH, a reversible, defined movement can be induced and the film turned to its initial flat shape again (Fig. 6). The process could be repeated reversibly in response to the alteration of surrounding conditions, showing a shape-memory property (Movie SM4 & SM5). However, the alteration of only one parameter by changing only temperature or humidity did not cause any significant responsive movements.

**Figure 7:**
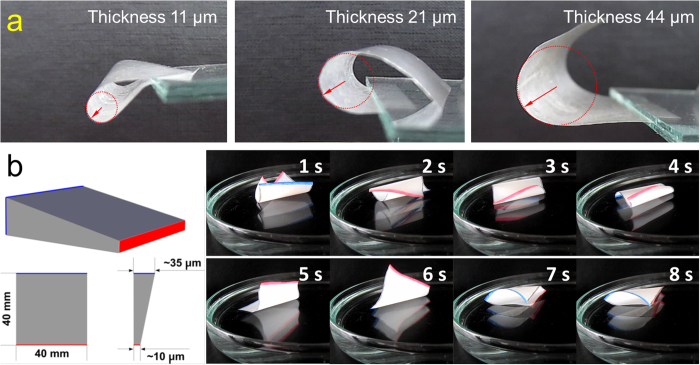
(**a**) Photographs of CSE_0.3_ films (40 × 40 mm^2^) with thicknesses of 10.9 ± 0.6, 21.2 ± 1.6 and 44.1 ± 3.5 μm in the state of maximal bending. The red arrows are visualizing the corresponding bending radii. (**b**) CSE_0.3_ film with a thickness gradient from 10.3 ± 1.1 μm at the one side (marked in red) to 34.7 ± 2.2 μm at the other side (marked in blue). Right panel shows the snapshots of the CSE_0.3_ film (40 × 40 mm^2^) coated with CSE_3_ NPs on water surface at RT (Movie SM6).

In addition to the alteration of the surface hydrophobicity of CSE_0.3_ films, the thicknesses of CSE_0.3_ films can also be modified. By increasing the film thickness, the minimal bending radius rises due to higher stiffness^10^,^48^. The radii at the maximal bending are measured to be 2.6, 4.6 and 9.6 mm for the CSE_0.3_ films with thicknesses of 10.9 ± 0.6, 21.2 ± 1.6 and 44.1 ± 3.5 μm, respectively (Fig. 7a & S11). The different bending extents due to the thickness provide the possibility to adjust the movement of the films by simply modifying the film thickness. A CSE_0.3_ film with a thickness gradient was thus fabricated and transformed into non-wetting, moisture-responsive films after spray-coating with CSE_3_ NPs (Fig. 7b). In comparison to the random movement of CSE_0.3_ films with homogeneous thickness, the thinner side of CSE_0.3_ films with thickness gradient bends faster than the thicker side and initiates more often the bending of the film (Movie SM6). As shown in Movie SM6, the CSE_0.3_ films with a thickness gradient and non–wetting surface also demonstrated the reversible shape transition process on water surface.

## Discussion

Inspired by naturally occurring moisture-responsive events, novel moisture-responsive materials are promising candidates for the fabrication of functional devices, such as sensors and actuators^7^,^8^,^9^,^10^,^11^. A particular interesting point is that the moisture is a green resource and readily available in comparison to many other stimuli, such as magnetic field and UV light with specific wave lengths^49^. By using a stearoyl ester of sustainable cellulose with a low degree of substitution (DS) of 0.3 (CSE_0.3_), transparent, self-standing and moisture-responsive films were obtained after the solvent-casting. These films exhibited rhythmical bending movements and reversible shape alterations, when they are exposed to water vapor. For instance, even the humidity of human hands can be used as stimuli and result in opening of CSE_0.3_ films (Fig. 4e), which can be taken as a signal. As shown above, such bending of CSE_0.3_ films is caused by the swelling force formed by the transient absorption of water molecules at one film surface^47^. Hydrogen bonding should be formed between water molecules and hydroxyl groups at cellulose backbone. After the bending from an environment with high relative humidity to an environment with low relative humidity, the swollen film surface releases the water molecules, so that it falls to its initial state due to the gravity or similar forces on both film surfaces (Figs 3c and 4d).

Other cellulose-based materials have also been reported to show moisture-responsive property, such as paper^50^ and films of hydroxypropylcellulose^51^. However, paper could not reversibly bend or move and became totally wet due to its strong capability of absorbing water^50^. In contrast, films of hydroxypropylcellulose exhibited reversible motions and could release water molecules in an environment of low humidity^51^. In comparison to films of hydroxypropylcellulose which have high DS ascribed to hydroxypropyl groups, the DS of stearoyl groups in CSE_0.3_ is much lower (of only 0.3), in order to achieve similar properties. In addition to various characterizations of films from CSEs showing different DSs, i.e. CSE_0.3_, CSE_1.3_ and CSE_3_, it is shown in the present work that the content of stearoyl groups strongly affect the properties of films derived from them.

For instances, films of CSEs with higher DSs, such as CSE_1.3_ and CSE_3_, did not show significant moisture-responsiveness. However, the films of CSE_1.3_ and CSE_3_ were responsive to the temperature. By combining the film of CSE_0.3_ and CSE_3_, bilayer films with combined thermo- and moisture-responsiveness were further fabricated. Such films can curl up into tubes as well as turn flat by changing surrounding conditions. Thus, these bilayer films not only combine the properties of two different compounds, but also endow the constructed materials new perspectives for novel applications^52^. For instance, stimuli-responsive microsized tubes can be fabricated from polymer films under controlled rolling conditions, which can be further used as microsized jets^53^,^54^.

By spray-coating CSE_0.3_ films with CSE_3_ NPs, CSE_0.3_ films with non-wetting surfaces were obtained. As-prepared CSE_0.3_ films show continuous bending movements on water surface and reversible shape transition. They can be used for the fabrication of moisture-responsive sensors^8^ or generators for piezoelectricity^11^. Furthermore, a thickness gradient could be generated within CSE_0.3_ films, so that non-symmetric bending movements can be initiated. The presence of particular structures including patterned structures within films could be used for the shape transformation of soft materials^55^. Moreover, controlled movements of polymeric films can be achieved, in order to use them as sensors or actuators^56^.

Finally, cellulose is the most abundant sustainable material on earth. It is also biocompatible, biodegradable and non-toxic^20^. Thus, we not only foresee a wide application range for these moisture-responsive films, but also a positive impact on the environment.

## Methods

### Materials

Microcrystalline cellulose (MCC) with an average granule size of 50 μm and a DP_n_ of 270 as well as stearoyl chloride (90%) were bought from Sigma-Aldrich (Steinheim, Germany). Other chemicals are all of analytical grade and were used as received. A cellulose membrane of regenerated cellulose with a molecular weight cut-off of 3500 was received from Carl Roth GmbH & Co. (Karlsruhe, Germany).

### Homogeneous synthesis of CSEs with DSs of 0.3 and 1.3 (CSE_0.3_ and CSE_1.3_)

CSEs with low and intermediate DS were prepared under homogeneous conditions^28^. In brief, cellulose (1 g) was dispersed in *N,N*-dimethylacetamide (DMAc) (40 ml) and the mixture was stirred at 130 °C for 30 min. Then, LiCl (3 g) was added and the system was purged with nitrogen. Under continuous stirring the suspension was allowed to cool down to room temperature (RT) overnight, leading to a clear solution. Thereafter, the temperature of the solution was raised to 60 °C, before stearic acid chloride and pyridine were added. After 3 h reaction at 60 °C, the warm reaction mixture was poured into 250 ml ethanol. The product was collected by centrifugation, purified by repeated precipitation in ethanol and dissolution in hot DMSO for CSE_0.3_ or tetrahydrofuran (THF) for CSE_1.3_, respectively.

### Heterogeneous synthesis of CSE with DS of 3 (CSE_3_)

CSE with DS of 3 was prepared according to previous reports with some minor modifications^44^. Typically, cellulose (1 g) was washed with methanol and pyridine to remove traces of moisture before it was suspended in 30 ml pyridine. Then, the mixture was heated to 100 °C under stirring. Stearic acid chloride (13.83 ml, 6 mol/mol AGUs) was added in drops to the hot suspension while the system was purged with nitrogen. After 1 h stirring at 100 °C, the hot reaction mixture was poured into 200 ml ethanol. The precipitate was separated by centrifugation and purified by repeated dissolution in dichloromethane as well as precipitation in 5 volumes ethanol, before it was dried at RT.

### Film formation

For the film formation, CSEs were dissolved in a proper solvent at a concentration of 10 mg/ml. The chosen solvents were toluene (CSE_3_), THF (CSE_1.3_) and DMSO (CSE_0.3_). The CSE solution was then pipetted into a petri dish at an amount of ~0.23 ml/cm^2^ and was allowed to dry. To achieve homogeneous films, the temperature was increased to 35 °C for THF, 55 °C for toluene and 90 °C for DMSO. These temperatures correspond to approximately half of the boiling point of each solvent. For the formation of films from CSE_0.3_ with a thickness gradient, the petri dishes were tilted at an angle of 3° during drying process. For the formation of CSE_0.3_/CSE_3_ bilayer films, a precast CSE_0.3_ film was covered with CSE_3_ solution in toluene and dried as described before.

### Formation of nanoparticles (NPs) from CSE_3_ and spray-coating onto CSE_0.3_ films

CSE_3_ NPs were formed via nanoprecipitation by dropping the dichloromethane solution of CSE_3_ (10 mg/ml) into 10 volumes ethanol under ambient conditions as described before^44^. Then, the NPs suspensions were concentrated to about 25 mg/ml by centrifugation and spray-coated onto CSE_0.3_ films using an airbrush gun (Harder & Steenbeck GmbH & Co. KG, Norderstedt, Germany).

## Additional Information

**How to cite this article**: Zhang, K. *et al*. Moisture-responsive films of cellulose stearoyl esters showing reversible shape transitions. *Sci. Rep*. **5**, 11011; doi: 10.1038/srep11011 (2015).

## Supplementary Material

Supplementary Information

Supplementary Movie S1

Supplementary Movie S2

Supplementary Movie S3

Supplementary Movie S4

Supplementary Movie S5

Supplementary Movie S6
