# Supplementary material for: Moisture-responsive films of cellulose stearoyl esters showing reversible shape transitions
Source: Sci Rep. 2015 Jun 8;5:11011. doi: 10.1038/srep11011 (PMC4458881; doi:10.1038/srep11011)
Supplement: Supplementary Information [file srep11011-s1.pdf]

Supplementary Information

# Moisture-responsive films of cellulose stearoyl esters showing reversible shape transitions

Kai Zhang,[a,]* Andreas Geissler,[a] Michaela Standhardt,[b] Sabrina Mehlhase,[a] Markus Gallei,[a] Longquan Chen,[c] Christina Marie Thiele[b]

[a] Ernst-Berl-Institute for Chemical Engineering and Macromolecular Science, Technische Universität Darmstadt, Alarich-Weiss-Str. 8, D-64287 Darmstadt, Germany
[b] Clemens-Schöpf-Institut für Organische Chemie und Biochemie, Technische Universität Darmstadt, Alarich-Weiss-Str. 16, D-64287 Darmstadt, Germany
[c] School of Physical and Mathematical Science, Nanyang Technological University, 21 Nanyang Link, Singapore 637371

* Corresponding author.
Tel.: +49 6151 1675831; Fax: +49 6151 162479
Email: zhang@cellulose.tu-darmstadt.de

## Content

*Movies:*

**Movie SM1**. Moisture-responsive $CSE_{0.3}$ film (speed ×4).

**Movie SM2**. Cellulose membrane (speed ×4).

**Movie SM3**. A $CSE_{0.3}$ film with homogeneous thickness coated with $CSE_3$ NPs floating and moving on water surface at 22°C in the air (speed ×4).

**Movie SM4**. Rolling of a bilayer film consisting of $CSE_3$ and $CSE_{0.3}$ layers during cooling down from 55°C to 22°C (speed ×8).

**Movie SM5**. Turning-flat of a bilayer film consisting of $CSE_3$ and $CSE_{0.3}$ layers during heating from 22°C to 55°C (speed ×8).

**Movie SM6**. A $CSE_{0.3}$ film with a thickness gradient coated with $CSE_3$ NPs floating and moving on water surface at 22°C in the air (speed ×4).

*Supplementary methods and results:*

**Part 1.** Synthesis and characterization of cellulose stearoyl esters (CSEs).

**Part 2.** Fabrication and characterization of CSEs films.

**Part 1. Synthesis and characterization of cellulose stearoyl esters (CSEs).**

**Methods**

**Elemental analysis**

The contents of carbon, hydrogen and nitrogen were determined with Elemental Analyser vario EL III CHN from Elementar (Hanau, Germany). The total degree of substitution ascribed to stearoyl groups ($DS_{SE}$) was calculated as reported before.[1]

**FTIR spectroscopy**

FTIR spectroscopy was conducted on Spectrum One FTIR Spectrometer (PerkinElmer, Massachusetts, USA) at RT between 4000 and 600 $cm^{-1}$ with a resolution of 4 $cm^{-1}$. The carefully dried samples were measured twice per 16 scans and an average spectrum was then generated for each sample. Baseline correction was conducted using the method 'concave rubber band algorithm' with 200 baseline points and 5 iterations using OPUS 6.5 (Bruker Optics GmbH, Ettlingen, Germany).

**Size exclusion chromatography (SEC)**

$CSE_3$ and $CSE_{1.3}$ were measured with THF as eluent on a system composed of a Waters 515 pump (Waters, Milford, CT), a TSP AS100 auto sampler, a Waters column oven, a Waters 486 UV detector operating at 254 nm, a Waters 410 RI-detector, and a DAWN DSP light scattering detector (Wyatt Technology, Santa Barbara, CA). For data acquisition and evaluation of the light-scattering experiments, Astra Ver. 4.73 (Wyatt Technology, Santa Barbara, CA) was used. The light-scattering instrument was calibrated using pure toluene, assuming a Rayleigh ratio of $9.78\times10^{-6}$ $cm^{-1}$ at 690 nm. A column set composed of SDV 1000, SDV 100000 and SDV 1000000 (PSS Polymer Standards Service GmbH, Mainz, Germany) was used for SEC analysis. An injection volume of 118 µL, a sample concentration of 1-2 g/l, a column temperature of 35°C, and a THF flow rate of 1 ml/min were used. $CSE_{0.3}$ was measured in *N,N*-dimethylformamide (DMF)/LiCl solution on a SECcurity GPC system consisting of a pump, an autosampler, a RI-detector of Agilent 1200 Series (Waldbronn, Germany) with a set of columns consisting of PSS Gram 5 and PSS Gram 1000 column. An injection volume of 100 µl of $CSE_{0.3}$ solution in DMF/LiCl at the concentration of 3 g/l was measured with the column temperature of 25°C and the solvent flow of 0.5 ml/min.

**NMR spectroscopy**

All NMR experiments were recorded on a Bruker AvanceIII 600 spectrometer at resonance frequencies of 600.4 ($^{1}H$) and 150.98 MHz ($^{13}C$) with a Triple resonance Broadband Inverse probe equipped with a Z-gradient (5 mm TBI 1H/31P/D-BB Z-GRD). The temperature-control was achieved with a Bruker BCU-X and was set to 330 K. All spectra were recorded without sample spinning. The assignment was performed using $^{13}C$, COSY, HSQC and HMBC spectra, which are available in the Bruker pulse sequence library. The following concentrations were used for the NMR measurements: 130 mg/mL of $CSE_3$ in benzene-$d_6$, 100 mg/mL of $CSE_{1.3}$ in Pyridine-$d_5$ and 50 mg/mL of $CSE_{0.3}$ in dimethyl sulfoxide-$d_6$ (DMSO-$d_6$). All $^{13}C$ spectra were recorded with TD = 51200 points, SW = 190 ppm, NS = 4096 and a relaxation delay of 2 s. All COSY spectra were recorded with $TD(f_2)$ = 2048 points, $TD(f_1)$ = 128 points, $SW(f_2)$ = 13 ppm, $SW(f_1)$ = 13 ppm, NS = 64 and a relaxation delay of 2 s. All HSQC spectra were recorded with $TD(f_2)$ = 1200 points, $TD(f_1)$ = 256 points, $SW(f_2)$ = 10 ppm, $SW(f_1)$ = 180 ppm, NS = 64, a relaxation delay of 1 s and INEPT-delays were optimized for 145 Hz coupling constants. All HMBC spectra were recorded with $TD(f_2)$

= 4096 points, TD(f1) = 256 points, SW(f2) = 10 ppm, SW(f1) = 180 ppm, NS = 256, and a relaxation delay of 1 s. The delays were optimized for 8 Hz long-range coupling evolution.

**Differential scanning calorimetry (DSC)**

DSC measurements were done on a computer-aided Mettler Toledo DSC1 (Mettler-Toledo, Gießen, Germany) between -20 and 100°C with a heating rate of 10 K/min. Dried nitrogen gas was purged with a constant flow rate during the measurement. The temperature reading and caloric measurements were calibrated using indium and tin as standards.

## Results

CSEs were synthesized according Scheme S1.

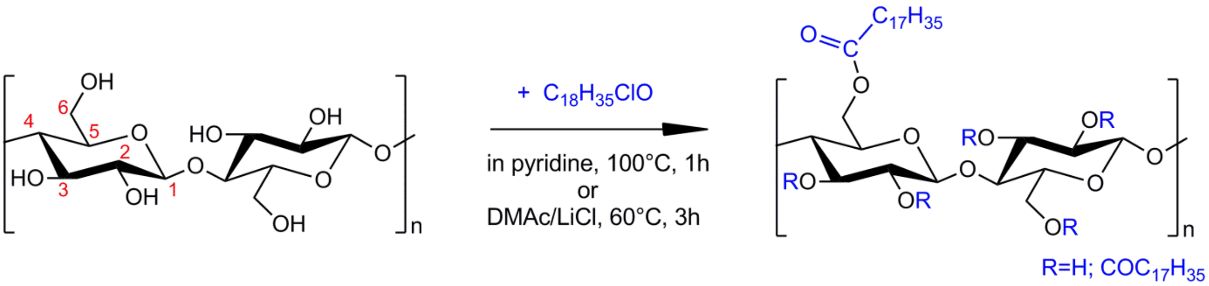


**Scheme S1**. Schematic illustration for the synthesis of CSEs with distinct DSs via two different synthesis routes.

**FTIR spectroscopy**

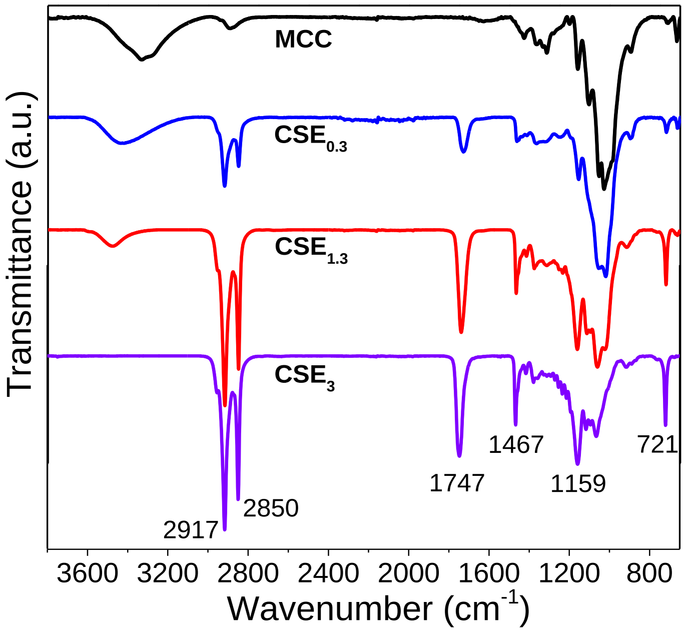


**Figure S1.** FTIR spectra (3800-650 cm$^{-1}$) of microcrystalline cellulose (MCC), $CSE_{0.3}$, $CSE_{1.3}$ and $CSE_3$ at RT.

FTIR spectra of synthesized CSEs showed typical signals attributed to aliphatic chains and ester bonds (Figure S1). The bands at 2917 and 2850 cm$^{-1}$ are derived from asymmetric and symmetric C-H stretching vibrations of methylene groups.[2] The bands at 1465 and 718 cm$^{-1}$

are attributed to the deformation and rocking vibrations of C-C groups of long aliphatic chains. The band at 1745 $cm^{-1}$ is ascribed to stretching vibrations of C=O groups, while the ester C-O-C stretching vibrations occur at 1158 $cm^{-1}$.[3-5]
All signals attributed to stearoyl groups exhibit increasing intensities with increasing DS, while the intensity of the FTIR signal attributed to stretching vibrations of hydroxyl groups decreases and the peak maximum is shifted to higher wavenumber. For $CSE_3$ with a DS of 3, the signals due to vibrations of hydroxyl groups cannot be observed anymore. The shifting of the OH signal with increasing DS (for CSE synthesized under homogeneous conditions) can be explained by the preferred substitution of the primary hydroxyl group and therefore with a favored disintegration of the $O(6)H\cdots O(3)$ intermolecular hydrogen bonds which can be observed in the wavelength range of 3230-3310 $cm^{-1}$.[6, 7] The intramolecular hydrogen bonds $O(3)H\cdots O(5)$ and $O(2)H\cdots O(6)$ occurring in the range of 3340-3375 $cm^{-1}$ and 3410-3460 $cm^{-1}$ were deconstructed, with the use of more stearic acid chloride leading to CSE with higher DS.[8]

**Solubility parameters**
The calculation of polymer solubility parameters was done according to Hoy.[9]

**Table S1.** Values of increments for the molar attraction function.[9]

| Structure group | $F_{t,i}[(MJ/m^3)^{1/2}/mol]$ | $F_{p,i}[(MJ/m^3)^{1/2}/mol]$ | $\Delta_{T,i}^{(P)}$ | $V_i[cm^3/mol]$ |
|---|---|---|---|---|
| **-$CH_3$** | 303.5 | 0 | 0.022 | 21.55 |
| **-$CH_2$-** | 269 | 0 | 0.020 | 15.55 |
| **>CH-** | 176 | 0 | 0.013 | 9.56 |
| **-COO-** | 640 | 528 | 0.050 | 23.7 |
| **-O- $_{acetal}$** | 236 | 102 | 0.018 | 6.45 |
| **-OH $_{prim}$** | 675 | 675 | 0.049 | 12.45 |
| **-OH $_{sec}$** | 591 | 591 | 0.049 | 12.45 |
| **ring** | -48 | 61 | -0.0035 | - |

**Table S2.** Summarized molar attraction functions for cellulose stearic acid esters (CSE) with different degree of substitution (DS).

| | $F_t[(MJ/m^3)^{1/2}/mol]$ | $F_p[(MJ/m^3)^{1/2}/mol]$ | $\Delta_T^{(P)}$ | $V[cm^3/mol]$ |
|---|---|---|---|---|
| **$CSE_3$** | 17315.5 | 1849 | 1.2935 | 958.4 |
| **$CSE_{1.3}$** | 9399.5 | 1956.1 | 0.7104 | 479.7 |
| **$CSE_{0.3}$** | 4621.8 | 2077.9 | 0.3674 | 198.1 |

**Equation S1.**
Molecular aggregation number.

$$\alpha(P) = 777\Delta_T^{(P)}/V \qquad (1)$$

**Equation S2.**
Number of repeating units per effective chain segment.

$$n = 0.5/\Delta_T^{(P)} \qquad (2)$$

**Equation S3.**

Total solubility parameter: ($\delta_t \,\hat{=}\, \delta$ )

$\delta_t = (F_i + \frac{B}{\bar{n}})/\,V$ (3), where the base value $B = 277.$

**Equation S4-6.**

Solubility parameters $\delta_d$ ,$\delta_p$ and $\delta_h$ which represent the influence of London dispersion forces (Van der Waals forces), polar forces and hydrogen bonds, respectively.

$$\delta_d = \left(\delta_t^2 - \delta_p^2 - \delta_h^2\right)^{1/2} \quad (4)$$

$$\delta_p = \delta_t\left(\frac{1}{\alpha^{(P)}}\frac{F_p}{F_t + B/\bar{n}}\right)^{1/2} \quad (5)$$

$$\delta_h = \delta_t\left[\left(\alpha^{(P)} - 1\right)/\alpha^{(P)}\right]^{1/2} \quad (6)$$

**Table S3.** Calculated solubility parameters for CSEs and those of the chosen solvents for the NMR measurements.[a]

| | $\boldsymbol{\delta_d}$[b] | $\boldsymbol{\delta_p}$[b] | $\boldsymbol{\delta_h}$[b] | $\boldsymbol{\delta}$ |
|---|---|---|---|---|
| $CSE_3$ | 17.40 | 5.88 | 4.05 | 18.81 |
| *Benzene* | *18.3* | *1.02* | *2.05* | *18.44* |
| $CSE_{1.3}$ | 17.03 | 8.50 | 7.39 | 20.42 |
| *Pyridine* | *18.92* | *8.79* | *5.93* | *21.69* |
| $CSE_{0.3}$ | 15.31 | 13.32 | 13.48 | 24.36 |
| *DMSO* | *18.41* | *16.36* | *10.23* | *26.67* |

[a] Solubility parameters of solvents were adapted from Hansen.[10]

[b] Solubility parameter components $\delta_d$, $\delta_p$ and $\delta_d$ represent the influence of London dispersion forces (van der Waals forces), dipole forces and hydrogen bonds, respectively.

## $^{13}C$ NMR spectroscopy

In comparison to widely used $^1H$ NMR and solid-state $^{13}C$ CP/MAS NMR spectroscopy, liquid-state $^{13}C$ NMR and 2D NMR spectra of long chain fatty acid esters of cellulose with low and intermediate DS are scarcely performed in contrast to cellulose esters with short alkane chains (< 6 carbons).[3, 11-14]

For the liquid-state NMR analysis, suitable solvents should be chosen whose signals do not affect the analysis or overlap with the signals of CSEs. The solubility of CSE is tightly dependent on the properties including the character of the functional group, its content referred as DS, and the molecular weight. Because the molecular weights of the CSEs are in the same order of magnitude (Figure 1b), the major difference is the content of stearoyl groups. Based on the chemical structure, the polymer solubility can be estimated based on solubility parameters. These parameters were calculated according to Hoy's increment method (Table S1-S3). The overall polarity of CSE decreases with increasing DS, due to the presence of non-polar stearoyl moieties. Thus, following solvents with similar solubility parameters were chosen for the liquid-state NMR measurements: benzene-$d_6$ for $CSE_3$, pyridine-$d_5$ for $CSE_{1.3}$ and DMSO-$d_6$ for $CSE_{0.3}$.

In general, the $^{13}C$ NMR spectra of the CSEs with different DSs recorded in different solvents are comparable, regarding the chemical shifts of carbons within AGU of cellulose and aliphatic chains. Nevertheless, small differences in chemical shifts ascribed to carbons of cellulose can be observed while the chemical shifts of aliphatic chains are not significantly affected by the solvents.

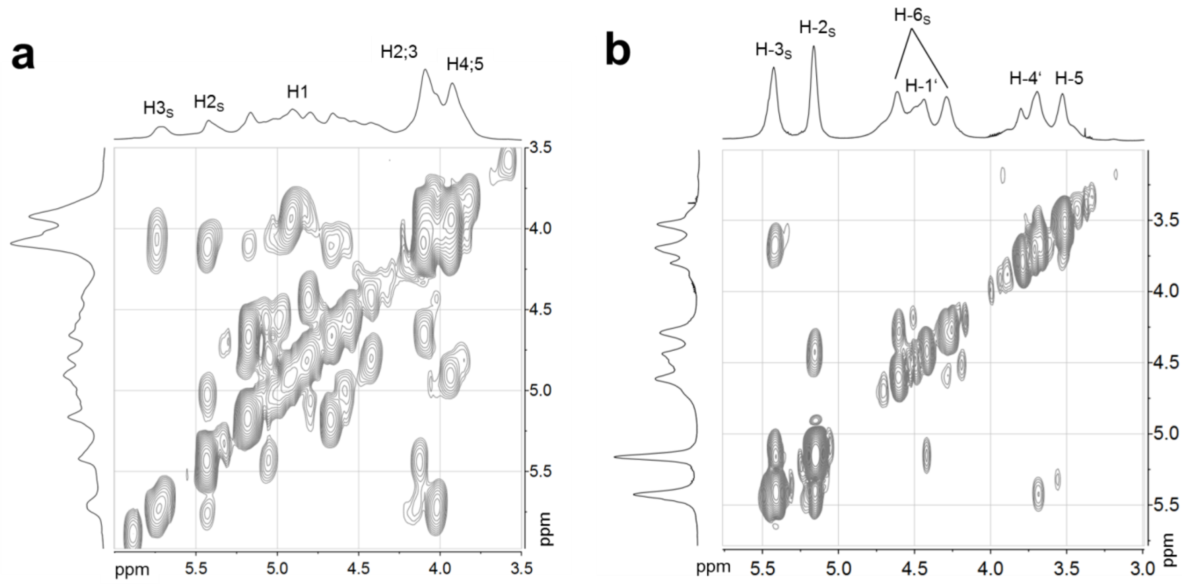


**Figure S2.** $^1H$, $^1H$-COSY NMR spectra (AGU-region) of (a) $CSE_{1.3}$ recorded in pyridine-$d_5$ at 50°C and (b) $CSE_3$ recorded in benzene-$d_6$ at 50°C. The subscript 's' indicates a chemical shift caused by substitution of the corresponding hydroxyl group, while an apostrophe denotes the substitution at a neighboring position.

**Table S4.** Chemical shifts (AGU and carbonyl) of $^{13}C$ NMR spectra depicted in Figure 1c.

| | $CSE_{0.3}$ | $CSE_{1.3}$ | $CSE_3$ |
|---|---|---|---|
| C6 | 60.2 | - | - |
| $C6_S$[a] | 62.5 | 63.6 | 63.2 |
| C2, C3, C5 | 74.7-72.9 | 76-73.4 | 73.8-72.7 |
| C4´ | - | 80.9 | 77.5 |
| C4 | 79.9 | 81.9 | - |
| C1´ | - | 101.5 | 101.6 |
| C1 | 102.5 | 104.2 | - |
| $C=O_{@C2}$ | - | 172.1 | 171.9 |
| $C=O_{@C3}$ | - | 172.8 | 172.2 |
| $C=O_{@C6}$ | 172.4 | 173.1 | 173.0 |

[a] The subscript „s“ indicates a chemical shift caused by substitution of the corresponding hydroxyl group, while an apostrophe denotes the substitution at a neighboring position.

In contrast to similar signals in the NMR region of aliphatic chains, the NMR regions ascribed to the carbons and hydrogens within the AGUs show altered chemical shifts attributed to substitutions at distinct positions. In order to elucidate the connectivity via hetero-nuclear $^1J$ and $^2J/^3J$ couplings between neighbored carbons and protons, 2D heteronuclear single-quantum correlation spectroscopy (HSQC) and heteronuclear multi-bond correlation spectroscopy (HMBC) were applied (Figure S3 & S4).
The HSQC and HMBC spectra of $CSE_{1.3}$ and $CSE_3$ depicted in Figure S3 & S4 reveal the coupling pathways within the aliphatic chains and AGUs. Due to the similarity of the aliphatic spectral regions, this section of the spectra is only presented once. $CSE_{0.3}$ was chosen as a representative for all stearic acid esters (Figure S4a). The AGU regions, which are framed by dotted lines, are described in detail.

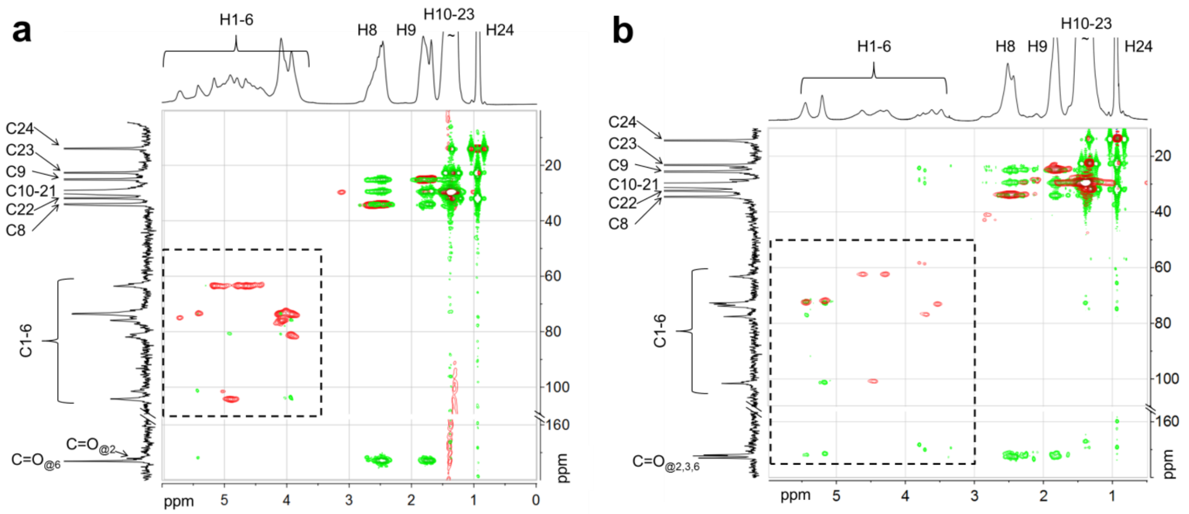


**Figure S3.** Overlaid HSQC (red) and HMBC (green) spectra of (a) $CSE_{1.3}$ recorded in pyridine-$d_5$ at 50°C and (b) $CSE_3$ recorded in benzene-$d_6$ at 50°C. The region which is presented in the main text is marked by the dotted lines.

Within the NMR spectra of $CSE_{0.3}$, several HSQC cross peaks corresponding to proton signals between 3-4.5 ppm can be observed (Figure S4b). These signals are attributed to hydrogen atoms bound to C1-C6. Separate signals were obtained for all of them except the C3 and C5 position, which are generally difficult to be distinguished. Due to the low DS of 0.3 and the preferred substitution of the primary hydroxyl group only one shifted carbon signal (62.5 ppm) attributed to $C6_S$ and the corresponding diastereotopic protons are visible: ($H6_1$; $C6_S$) and ($H6_2$; C6s).
In the range of 4.5-5.5 ppm, proton signals were observed which do not show cross peaks in HSQC spectrum. Therefore, these peaks are rather to be assigned to hydrogens in hydroxyl groups, instead of hydrogens bound to carbons. Three signals are visible at 4.5, 4.59 and 5.18 ppm, which can be assigned based on their HMBC cross peaks (Figure S4b). In opposite to other solvents, hydroxyl-proton couplings have been observed in DMSO, due to the reduced proton exchange rate.[15] Moreover, it is notable that all possible $^2J$ and $^3J$ coupling signals of these hydroxyl-protons can be found in the spectrum. The hydroxyl group hydrogen at C2 position couples with AGU carbons C1, C2 and C3, while the hydrogen at C3 position shows coupling to the carbons C2, C3 and C4. The proton of the primary hydroxyl group couples with the backbone carbons C5 and C6. Moreover, the (H1; C4+) long range coupling can be observed.

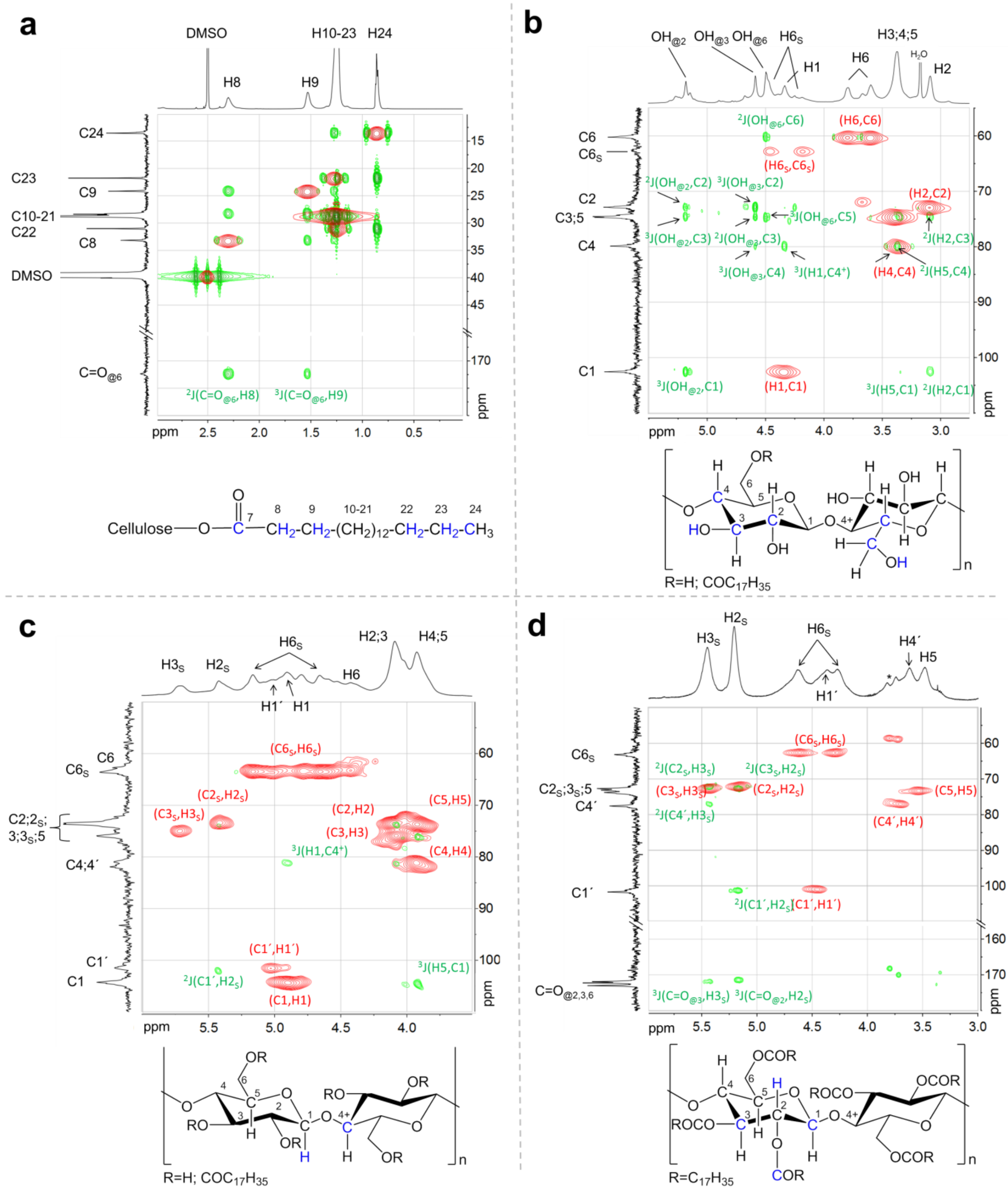

**Figure S4.** Overlaid HSQC (red) and HMBC (green) spectra of CSEs. (a) The aliphatic region and (b) the AGU region of $CSE_{0.3}$, recorded in DMSO-$d_6$ at 50°C. (c) The AGU region of $CSE_{1.3}$ recorded in pyridine-$d_5$ at 50°C. (d) The AGU region of $CSE_3$ recorded benzene-$d_6$ at 50°C. The chemical sketch of each CSE is shown below the corresponding 2D spectra for the visualization of $^2J$ and $^3J$ couplings (marked in blue). The subscript „s“ indicates a chemical shift caused by the substitution of the corresponding hydroxyl group, while an apostrophe denotes the substitution at a neighboring position.

The HSQC and HMBC spectrum of $CSE_{1.3}$ are depicted in Figure S4c. Due to signal splitting, caused by increased substitution of hydroxyl groups, the NMR spectra of $CSE_{1.3}$ become complicated. Thus, $^1H$, $^1H$-COSY was also measured for the peak assignment (Figure S2a). It is notable that substitution of primary hydroxyl groups is almost complete. A weak HSQC

signal is visible at ~ 61.5 ppm, which can be assigned to ($H6_1$; C6) and ($H6_2$; C6) and therefore to non-substituted primary hydroxyl groups. A broad cross peak at ~ 63.6 ppm represents the coupling to the diastereotopic protons: ($H6_1$; $C6_S$) and ($H6_2$; C6s). Furthermore, a weak substitution of secondary hydroxyl groups can be detected. Due to the substitution, the protons at C2 and C3 position were deshielded by 1.5 ppm. Therefore, cross peaks at 5.42 and 5.72 ppm in HSQC spectrum indicate the esterification of C2 and C3 position, respectively. Furthermore, HMBC crosspeaks for $H2_S$@C=O and $H3_S$@C=O can be observed. The chemical shift in $^{13}$C-NMR spectrum, induced by the substitution at the C2 and C3 position is not significant, so that the signals cannot be distinguished directly. Due to changes in the chemical environment by the substitution of $OH_{@2}$, the H1' and C1' splitting signals appear as an indirect sign of C2 substitution. Furthermore, the $^3$J couplings (H5; C1) across the AGU ring oxygen as well as (H1; C4+) across the glycosidic linkage were detected within the HMBC spectrum of $CSE_{1.3}$ (Figure S4c).
As shown in Figure S4d, the AGU region within the HSQC spectrum of $CSE_3$ contains seven signals attributed to seven hydrogen atoms of the 2,3,6-O-substituded AGUs. Signal splitting due to partial substitution does not exist anymore. Within the HMBC spectrum, six cross peaks related to substituted hydroxyl groups at C2 and C3 position dominate. It is possible to distinguish the coupling signals ($H_3$; $C_2$/$C_4$/C=$O_{@3}$) as well as ($H_2$; $C_1$/$C_3$/C=$O_{@2}$) as shown at 5.4 and 5.2 ppm for $H3_S$ and $H2_S$. In particular, the long range coupling across the ester oxygen can be significantly detected for the position C2 and C3. Three C=O signals were observed for $CSE_3$, which can be attributed to ester bonds at the position C2, C3 and C6 at 171.9; 172.2 and 173 ppm, respectively.[16] The signal caused by the esterified primary hydroxyl group appears most deshielded (furthest low field). The signals for C=$O_{@3}$ and C=$O_{@2}$ are shifted slightly to higher field. In contrast to CSEs with lower DS, HMBC of $CSE_3$ does not show $^3$J coupling across the ring oxygen or the gycosidic linkage.

## Part 2. Fabrication and characterization of CSEs films.

### Methods

### Ellipsometry

Ellipsometry was conducted on Ellipsometry System $EP^3$ (Accurion GmbH, Göttingen, Germany) in air to determine the thickness change of CSEs films dip-coated on silicon wafers during the temperature increase. The laser (He-Ne laser) with a wave length of 658 nm was used and the data were collected in 1.0° intervals between 40 and 80°. At least three regions on film surfaces were measured and obtained values were presented as the average values with standard deviations. A three phase optical model as ambient-film-silicon substrate was used for the calculation of film thickness and refractive index. A multilayer system consisting of silicon, $SiO_2$, cellulose derivatives and air was established using analytical program - EP4 Model software. Refractive indexes of Si and $SiO_2$ were adapted as reported before.[17]. The thickness of the $SiO_2$ layer was determined in triplicate and averaged to 2.7 nm. The thickness of the silicon was presumed as infinite. After the base layers were defined, the thickness and the refractive index of the unknown layer of cellulose esters were calculated by fitting the obtained curves through a “global fit mode”-function of the analyzing program.

### Thickness measurement

The thickness of the CSE films was measured using a micrometer caliper (Precise PS 7230, Burg Wächter) with a precision of 1 µm. In general, the films were measured site-related with 1 measurement per $cm^2$ for the calculation of the average values, which means 16 measurements for a 40x40 $mm^2$ sized sample.

### UV-Vis spectroscopy

The transmittance measurements of the films were performed in triplicate using an UV-Vis spectrometer (Cary 60, Agilent Technologies). The 20 µm thick samples were measured with a scan rate of 300 nm/min over a wavelength range of 1100-200 nm at RT.

### Mechanical properties

The tensile strength measurements were performed at 23±1°C and 50±2% relative humidity (RH) on a Zwick Z010 (Zwick Roell, Germany) equipped with a 200 N load cell. The initial grip distance was set to 50 mm and the tests were conducted with a strain rate of 25 mm/min. Before the measurements, the films of 20×70 $mm^2$ were equilibrated under these conditions for at least 24 h. For each film, 10 measurements were performed and the average results were presented.

### Static water contact angle measurement

Static contact angles were measured using a Contact Angle Goniometer DSA30 (Krüss, Hamburg, Germany) within a thermostatic chamber with the constant temperature of 23±1°C and the RH of 50±2%. A water droplet of 4 µl was placed onto the film while a video was recorded. Afterwards, the initial contact angles, as well as the ones after 30, 60 and 120 s were calculated using the processing software SCA20 (DataPhysics, Filderstadt, Germany).

### Static water vapor permeability (sWVP)

The water vapor permeability (sWVP) for CSEs films was determined gravimetrically as reported before with some modifications.[18] Before the measurements, the films were equilibrated at 23±1°C and 50±2% RH for 24 h. Then, the films were used to seal cups containing deionized water or anhydrous calcium chloride. The distance between the films and the surface of water or calcium chloride was set at 1 cm. The systems containing CSEs

films were stored at 23±1°C and 50±2% RH for another 72 h. The weights of samples were continuously weighted after distinct times. Then, the sWVP was calculated according to equation (7). All measurements were done in triplicate and average values were used for the calculation.

$$sWVP(g/(m*s*Pa)) = \frac{\Delta m * h}{A * \Delta t * \Delta P}$$ (7), where Δm is the weight change of the system after a storage time of Δt; A and h are the film area and the film thickness, respectively; ΔP represents the difference of the water vapor partial pressure between the two sides of the films.

**Scanning electron microscopy (SEM)**

SEM images were obtained on a Philips XL30 FEG high-resolution scanning electron microscope (HR-SEM) (FEI Deutschland GmbH, Frankfurt/Main, Germany). A layer of 10 nm platinum/palladium (80/20) was deposited on the sample surface to achieve electrical conductivity.

## Results

### Transparency

It has to be noted that the optical properties of the film are highly influenced by the surface roughness of the mold they were produced on. For the UV-Vis measurement, the films of all three CSEs were prepared on glass surface. For the comparison, the films of $CSE_{1.3}$ and $CSE_{0.3}$ prepared in PTFE-molds were shown in Figure S5. Due to slow solvent evaporation, the upper side of the film is smooth, while the lower side is forming an inversed pattern of the substrate surface within PTFE-molds. In comparison to films of $CSE_3$ produced within glass petri dishes, the films of $CSE_{1.3}$ and $CSE_{0.3}$ casted within PTFE petri dishes are due to the rough surface only translucent.

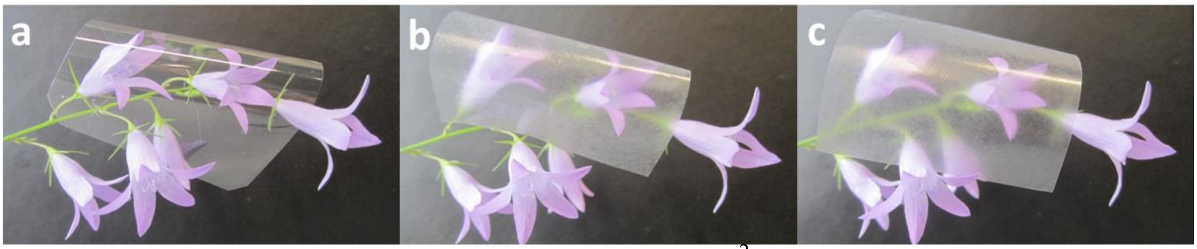


**Figure S5.** Photographs of films from CSEs (40x40 mm$^2$) with flowers beneath the films for the visualization. (a) Transparent $CSE_3$ film of 19 µm formed on smooth glass surface. (b) Translucent films of $CSE_{1.3}$ and (c) $CSE_{0.3}$ of 15 µm casted on PTFE surface.

**Scanning electron microscope (SEM)**

As shown in Figure S6, the membrane of regenerated cellulose shows the cross-section with a layered structure. The membrane of regenerated cellulose is transparent, as shown in the Movie S1.

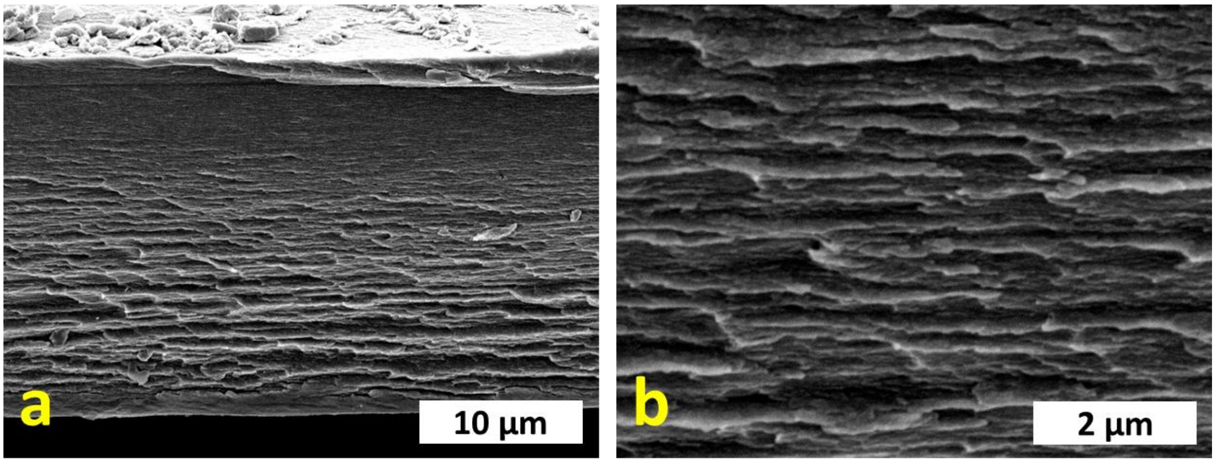


**Figure S6.** SEM images of the cross-section of a membrane from regenerated cellulose with the thickness of 24.3±1.2 µm (Carl Roth GmbH & Co., Karlsruhe, Germany) in two magnifications: (a) 5000 and (b) 25000 times.

**Moisture-responsive properties**

The property of films to absorb water strongly depends on the presence of free hydroxyl groups. The intensity of moisture absorbance is continuously decreasing with increasing DS. While a $CSE_{1.3}$ film (a-c) is still able to perform marginal movements, the $CSE_3$ film (d-f) stays stationary under equal conditions (Figure S7).

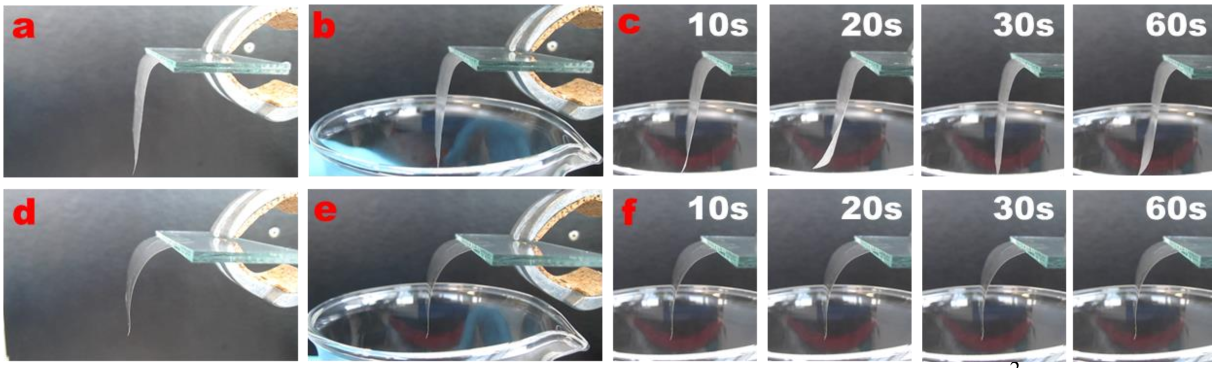


**Figure S7**. Snapshots of (a-c) a $CSE_{1.3}$ film and (d-f) a $CSE_3$ film (40x40 $mm^2$) with one side fixed between two glass slides. (b) $CSE_{1.3}$ film and (e) $CSE_3$ film after immediately placing a beaker with 37°C warm water underneath the film. (c and f): Snapshots of the films after distinct times.

To make sure that moisture and not heat (at 37°C of water) is the driving force for film movements, a reference test with $CSE_{0.3}$ using warm silicone oil of 37°C was performed. Under analogue experimental conditions, it is obviously that heat alone does not cause any response (Figure S8).

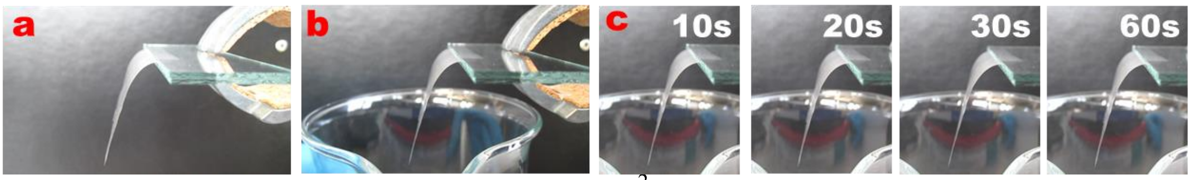


**Figure S8**. Snapshots of a $CSE_{0.3}$ film (40x40 $mm^2$) above a silicone oil bath of 37°C. (a) The initial state with one side fixed between two glass slides. (b) After placing the beaker with silicone oil of 37°C under the film. (c) Snapshots of the film after distinct times.

**Mechanical properties**

The tensile strength tests revealed the significant decrease of tensile properties which are accompanied with an increasing DS (Table S5). The $CSE_{0.3}$ film with 10% hydroxyl groups substituted by stearoyl moieties exhibited a strongly decreased elastic modulus which is only ~15% of that of regenerated cellulose.

**Table S5.** Mechanical properties of the membrane from regenerated cellulose and the films from CSEs.

| samples | Elastic modulus [MPa] | Tensile strength [N/mm$^2$] | Fracture strain [%] |
|---|---|---|---|
| Regenerated cellulose | 7230 ± 120 | 169.2 ± 8.1 | 15.9 ± 1.5 |
| $CSE_{0.3}$ | 1118 ± 86 | 28.5 ± 1.6 | 12.7 ± 1.7 |
| $CSE_{1.3}$ | 397 ± 52 | 10.4 ± 1 | 10.7 ± 2.3 |
| $CSE_3$ | 286 ± 54 | 5.5 ± 2.1 | 2.5 ± 1 |

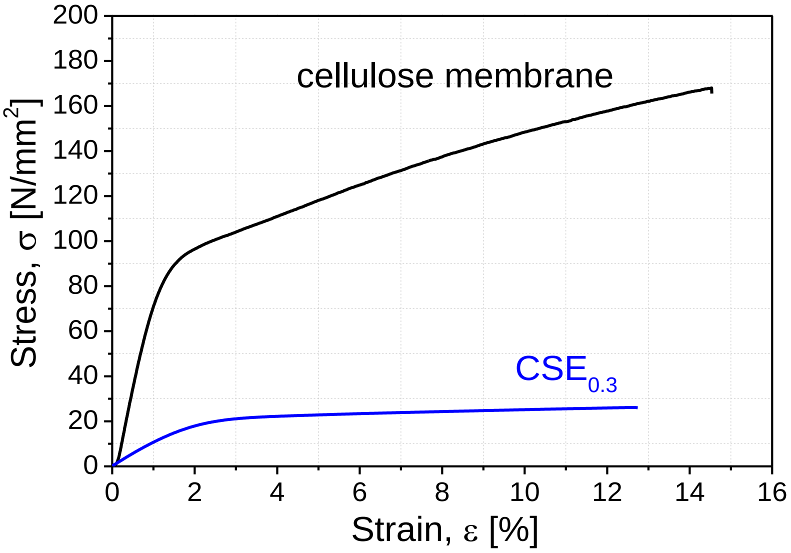


**Figure S9.** Representative stress-strain curves of a cellulose membrane from regenerated cellulose (Carl Roth GmbH & Co., Karlsruhe, Germany) with the thickness of 24.3±1.2 µm and a $CSE_{0.3}$ film of 21.2±1.6 µm. The longitudinal mechanical strength of the cellulose membrane was measured.

**Static water contact angle measurement**

$CSE_3$ film exhibited the highest initial contact angle of ~110° and the contact angle decreased to ~108° after 2 minutes. In comparison, the contact angles on $CSE_{1.3}$ and $CSE_{0.3}$ film are slightly lower and decreased to around 96° after 2 minutes.

**FTIR spectra of CSE films after the equilibration in the environments with diverse humidities**

After the equilibration under the surroundings with different relative humidities of 100%, 50% and ~0%, the films from $CSE_{0.3}$ contained different amount of water. In Figure S10, FTIR spectra also confirmed the presence of different contents of water within the films according to the intensity of the signals at 1635 $cm^{-1}$, which is ascribed to vibrations of water molecules.

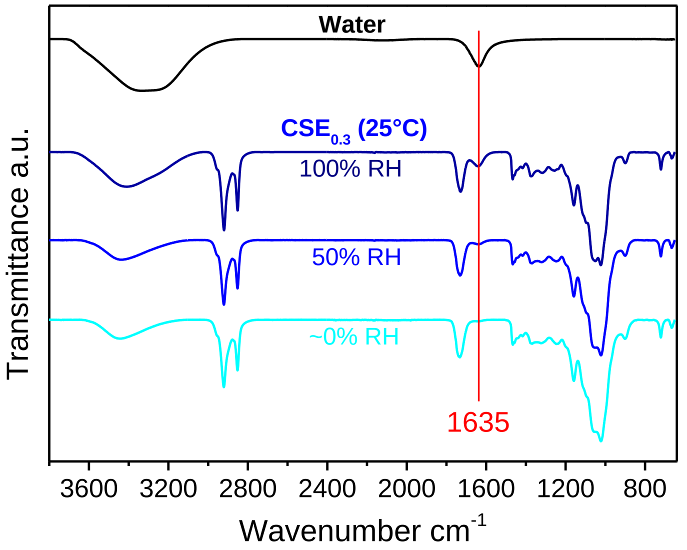


**Figure S10.** FTIR spectra (3800-600 $cm^{-1}$) of water and $CSE_{0.3}$ at 25°C under surroundings with different relative humidities (RH) of 100%, 50% and ~0%.

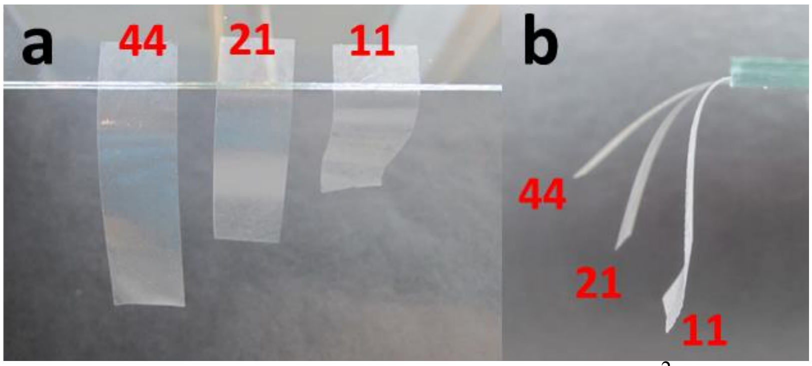


**Figure S11.** Photographs of $CSE_{0.3}$ films (10x40 $mm^2$) with different thicknesses of 10.9±0.6, 21.2±1.6 and 44.1±3.5 μm, respectively. (a) Top view and (b) side view of clamped films between two glass slides. The red numbers are thicknesses in μm.

## References


1. Vaca-Garcia, C., Borredon, M.E. & Gaseta, A. Determination of the degree of substitution (DS) of mixed cellulose esters by elemental analysis. *Cellulose* **8**, 225-231 (2001).
2. Jandura, P., Kokta, B.V. & Riedl, B. Fibrous long-chain organic acid cellulose esters and their characterization by diffuse reflectance FTIR spectroscopy, solid-state CP/MAS $^{13}$C-NMR, and X-ray diffraction. *J. Appl. Polym. Sci.* **78**, 1354-1365 (2000).
3. Fumagalli, M., Ouhab, D., Boisseau, S.M. & Heux, L. Versatile gas-phase reactions for surface to bulk esterification of cellulose microfibrils aerogels. *Biomacromolecules* **14**, 3246-3255 (2013).
4. Geissler, A., Chen, L., Zhang, K., Bonaccurso, E. & Biesalski, M. Superhydrophobic surfaces fabricated from nano- and microstructured cellulose stearoyl esters. *Chem. Commun.* **49**, 4962-4964 (2013).
5. Socrates, G. Infrared and Raman characteristic group frequencies, Edn. 3rd. (Wiley, England; 2001).
6. Liang, C.Y. & Marchessault, R.H. Infrared spectra of crystalline polysaccharides. I. Hydrogen bonds in native celluloses. *J. Polym. Sci.* **37**, 385-395 (1959).
7. Moon, R.J., Martini, A., Nairn, J., Simonsen, J. & Youngblood, J. Cellulose nanomaterials review: structure, properties and nanocomposites. *Chem. Soc. Rev.* **40**, 3941-3994 (2011).
8. Schwanninger, M., Rodrigues, J.C., Pereira, H. & Hinterstoisser, B. Effects of short-time vibratory ball milling on the shape of FT-IR spectra of wood and cellulose. *Vib. Spectrosc.* **36**, 23-40 (2004).
9. Krevelen, V. Properties of Polymers, Edn. 4. (Elsevier, Amsterdam; 2009).
10. Hansen, C.M. The Universality of the Solubility Parameter. *Ind. Eng. Chem. Prod. Res. Develop.* **8**, 2-11 (1969).
11. Berlioz, S., Molina-Boisseau, S., Nishiyama, Y. & Heux, L. Gas-phase surface esterification of cellulose microfibrils and whiskers. *Biomacromolecules* **10**, 2144-2151 (2009).
12. Buchanan, C.M., Hyatt, J.A. & Lowman, D.W. Two-Dimensional Nmr of Polysaccharides - Spectral Assignments of Cellulose Triesters. *Macromolecules* **20**, 2750-2754 (1987).
13. Fumagalli, M., Sanchez, F., Boisseau, S.M. & Heux, L. Gas-phase esterification of cellulose nanocrystal aerogels for colloidal dispersion in apolar solvents. *Soft Matter* **9**, 11309 (2013).
14. Kono, H., Erata, T. & Takai, M. CP/MAS13C NMR Study of Cellulose and Cellulose Derivatives. 2. Complete Assignment of the13C Resonance for the Ring Carbons of Cellulose Triacetate Polymorphs. *J. Am. Chem. Soc.* **124**, 7512-7518 (2002).
15. Moritani, T., Kuruma, I., Shibatani, K. & Fujiwara, Y. Tacticity of Poly(vinyl alcohol) Studied by Nuclear Magnetic Resonance of Hydroxyl Protons. *Macromolecules* **5**, 577-580 (1972).
16. Miyamoto, T., Sato, Y., Shibata, T., Inagaki, H. & Tanahashi, M. $^{13}$C Nuclear Magnetic Resonance Studies of Cellulose Acetate. *J. Polym. Sci. Part A Polym. Chem.* **22**, 2363-2370 (1984).
17. Kosaka, P.M., Kawano, Y., Salvadori, M.C. & Petri, D.F.S. Characterization of Ultrathin Films of Cellulose Esters. *Cellulose* **12**, 351-359 (2005).
18. Bras, J., Vaca-Garcia, C., Borredon, M.-E. & Glasser, W. Oxygen and water vapor permeability of fully substituted long chain cellulose esters (LCCE). *Cellulose* **14**, 367-374 (2007).
